# Improved Results for Resection of Periampullary Adenocarcinoma

**DOI:** 10.1155/1996/91791

**Published:** 1996

**Authors:** H. Obertop, D. J. Gouma

**Affiliations:** Department of Surgery Academic Medical Centre Meibergdreef 9 Amsterdam AZ 1105 The Netherlands

## Abstract

*Background*: This study evaluates the indications for and effects of pancreaticoduodenectomy (102 patients) or total pancreatectomy (15 patients) with extensive lymph node dissection performed upon 117 patients for treatment of periampullary adenocarcinoma.

*Study Design*: Presenting symptoms and postoperative morbidity and mortality rates were recorded. Cumulative survival rates were evaluated in relation to origin, size, and staging of tumor. Postoperative follow-up of clinical symptons was done after one year.

*Results*: The postoperative mortality rate after Whipple’s operation was 8 percent (eight patients). The median survival period was 1.1 year and the overall five year survival rate was 15 percent (confidence limits, 5 to 25 percent). The five year survival rate for patients without tumor extension beyond the pancreas was 25 percent (confidence limits, 5 to 50 percent), and in patients with adenocarcinoma of the ampulla of Vater, 34 percent (confidence limits, 3 to 65 percent). The median survival rate in patients with adenocarcinoma of the ampulla of Vater was 3.3 years, which was significantly longer than in the other patients. Fifty-nine patients with distant spread could be divided into 14 patients with para-aortic lymph node metastases who had a significantly shorter survival period than 45 patients without para-aortic lymph node metastases (p=0.004). Most patients surviving more than one year were doing well, although 60 percent needed exocrine pancreatic substitution therapy.

*Conclusions*: Resection of periampullary carcinoma provides a better palliation and survival rate than nonoperative biliary drainage or bypass operation. An improved preoperative verification of para-aortic metastases could restrict resection to patients with a prognostic five year survival rate of more than 25 percent and a postoperative mortality rate of less than 5 percent.


detrimental effect on hepatic regeneration was more due to portal pooling and reperfusion of portal blood than ischemia/reperfusion by itself.

One problem in the preoperative evaluation of patients with liver cirrhosis is how to estimate the amount of ischaemia that can be inflicted on the liver with no harm. The patient group with liver cirrhosis comprises a wide range of functional reserve in relation to operative tolerance to ischemia. This is particularly difficult since we do not know which cell types in the liver are more prone to hypoxia. One group of patients which seems to be at particularly high risk for morbidity is patients with cholestasis. In this group of patients with cirrhosis and cholestasis preoperative drainage prior to resection may be indicated.

In conclusion, patients with cirrhosis can undergo hepatic resection with inflow occlusion with reduced blood loss and thereby reduced morbidity without inflicting any significant ischaemic damage to the liver, if ischaemia time is of one hour duration or less.

### REFERENCES

1. Nishimura T, Nakahara M, Kobayashi S, Hotta I, Yamawaki S, Marui Y: (1992) Ischemic injury in cirrhotic livers: An experimental study of the temporary arrest of hepatic circulation. *Journal of Surgical Research* **53**: 227–233.
2. Nagorney DM, van Heerden JA, Ilstrup DM, Adson MA: (1989) Primary hepatic malignancy: Surgical management and determinants of survival. *Surgery* **106**: 740–749.
3. Nagasue N, Uchida M, Kubota H, Hayashi T, Kohno H, Nakamura T: (1995) Cirrhotic liver can tolerate 30 minutes ischaemia at normal environmental temperature. *European Journal of Surgery* **161**: 181–186.
4. Emond J, Wachs M, Renz J, Kelley S, Harris H, Roberts J, Ascher N, Lim R: (1995) Total vascular exclusion for major hepatectomy in patients with an abnormal liver parenchyma. *Archives of Surgery* **130**: 824–831.
5. Kim IY, Kobayashi M, Nakashima K, Aramahi M, Yoshida T, Mitarai Y: (1994) In situ and surface liver cooling with prolonged inflow occlusion during hepatectomy in patients with chronic liver disease. *Archives of Surgery* **129**: 169–624.
6. Usami M, Furuchi K, Shiroiwa H, Saitoh Y: (1994) Effect of repeated portal-triad cross-clamping during partial hepatectomy on hepatic regeneration in normal and cirrhotic rats. *Journal of Surgical Research* **57**: 541–548.

Bengt Jeppsson
Department of Surgery
Lund University Hospital
S-221 85 LUND
Sweden

### IMPROVED RESULTS FOR RESECTION OF PERIAMPULLARY ADENOCARCINOMA

### ABSTRACT

*Andersen, H.B., Baden, H., Brahe, N.E.B. and Burcharth, F. (1994) Pancreaticoduodenectomy for periampullary adenocarcinoma Journal of the American College of Surgeons 179: 545–552.*

***Background:*** **This study evaluates the indications for and effects of pancreaticoduodenectomy (102 patients) or total pancreatectomy (15 patients) with extensive lymph node dissection performed upon 117 patients for treatment of periampullary adenocarcinoma.**
***Study Design:*** **Presenting symptoms and postoperative morbidity and mortality rates were recorded. Cumulative survival rates were evaluated in relation to origin, size, and staging of tumor. Postoperative follow-up of clinical symptons was done after one year.**
***Results:*** **The postoperative mortality rate after Whipple's operation was 8 percent (eight patients). The median survival period was 1.1 year and the overall five year survival rate**

**was 15 percent (confidence limits, 5 to 25 percent). The five year survival rate for patients without tumor extension beyond the pancreas was 25 percent (confidence limits, 5 to 50 percent), and in patients with adenocarcinoma of the ampulla of Vater, 34 percent (confidence limits, 3 to 65 percent). The median survival rate in patients with adenocarcinoma of the ampulla of Vater was 3.3 years, which was significantly longer than in the other patients. Fifty-nine patients with distant spread could be divided into 14 patients with para-aortic lymph node metastases who had a significantly shorter survival period than 45 patients without para-aortic lymph node metastases (p=0.004). Most patients surviving more than one year were doing well, although 60 percent needed exocrine pancreatic substitution therapy.**

***Conclusions:*** **Resection of periampullary carcinoma provides a better palliation and survival rate than nonoperative biliary drainage or bypass operation. An improved preoperative verification of para-aortic metastases could restrict resection to patients with a prognostic five year survival rate of more than 25 percent and a postoperative mortality rate of less than 5 percent. *J. Am. Coll. Surg.,* 1994, 179: 545–552.**

KEY WORDS: Periampullary carcinoma Pancre scarcinoma Pancreatico-duodenectomy.

### PAPER DISCUSSION

Resection of the pancreatic head and adjacent structures offers the only hope for cure of patients with adenocarcinoma of the periampullary region consisting of cancer of the pancreatic head, ampulla of Vater, distal common bile duct and duodenum. Although these tumours are frequently lumped together because of their similar symptomatology and diagnostic and therapeutic approach, the prognosis after resection differs substantially as shown in the paper of Anderson and coauthors.[1] Especially for adenocarcinoma of the pancreatic head tumours, the possible benefit of resection is small and the costs expressed as morbidity can be substantial even though operative mortality ranges from 0 to 8.9% in the last few years[2–9]. In fact this operative mortality is even lower than after seemingly less invasive surgical procedure such as gastric resection for cancer[10]. The improved operative mortality after pancreatoduodenectomy in experienced hands has led to a more aggressive approach although long term survival has not improved much in patients surviving the resection[11] as compared to the days when Crile advised strongly against Whipple's procedure for pancreatic head cancers[2].

There is a strong correlation between surgical experience and operative risk[13,14]. Centres with a volume of more than eighty cases in an eight-year study period had a mortality of 5.5% as compared with 12.9% mortality in medium (51–80), 11.8% in low (10–50) and 18.9% in minimal (<10) volume hospitals[14]. According to these criteria the data in the paper by Andersen *et al.*[1], come from a low to medium volume hospital and the operative mortality of 12% is what can be expected. The mortality in 102 of the 117 patients undergoing pancreato-duodenectomy is 8%, but additional portal vein resection and/or total pancreatectomy led to a prohibitive high operative mortality in those 15 patients. These data should caution the authors, as well as other pancreatectomists, to reconsider the necessity of extensive procedures since there is little evidence that resections of major vessels such as the portal or mesenteric vein improves the longterm survival after pancreato-duodenectomy[15]. However, when the point of no return is passed, tumour can be left behind at the portal vein and a pancreato-duodenectomy performed for palliation. This can be done with low mortality and an acceptable quality of life[16].

Total pancreato-duodenectomy for cancer has been abandoned by most surgeons because evidence that this more extensive operation will improve survival is lacking[17]. For practical reasons many surgeons re-resect a part of the pancreatic remnant when frozen section shows tumor residue at the pancreatic resection margin.

Even in one series with a zero operative mortality after pancreato-duodenectomy, morbidity was present in about half of the patients[4]. Leakage of the pancreatico-enterotomy is one of the leading causes of postoperative morbidity in 5 to 25% of the patients in most series regardless of the type of anastomosis[18] or whether a pancreaticojejunostomy or pancreatico-gastrostomy is performed[19]. Perioperative administration of Somatostatin has been reported to lower the chance of this complication[20]. Andersen *et al.* [1] report anastomotic leakages at the pancreato-jejunostomy site in 9 patients, leading to postoperative death in 2 patients. A 25 % mortality after leakage of a pancreatic anastomosis has been reported by others[18]. Although anastomotic leakage even occurs in centres with a large volume and extensive experience, mortality due to this complication can be avoided by agressive postoperative monitoring, using mainly clinical signs and symptoms[21].

Although preoperative obstructive jaundice is a well known risk factor in pancreatic surgery[22], and although studies in experimental animals univocally stress the beneficial effect of preoperative drainage on immune status, endotoxaemia, kidney and liver function, wound healing and other parameters[23,24,25], the beneficial effect of preoperative biliary drainage in man has not been shown in prospective randomized trials. The possible complications of external or internal drainage may very well outweigh the possible benefits of the procedure[26,27]. Andersen *et al.*[1] report that 47 of 117 patients underwent pre-operative drainage but they do not elucidate the indication for this procedure. Their hypothesis that pre-operative biliary drainage would cause a technically more demanding biliodigestive anastomosis has neither been substantiated by the literature nor by our own experience.

It may well be that postoperative gastrointestinal and intra-abdominal bleeding, mentioned in the study by Andersen *et al.*[1] were the result of erosion of a major artery that is a well known complication of pancreatic and biliary surgery[28]. Andersen *et al.*[1] show a tendency to a higher postoperative morbidity and mortality rate among patients who were older than seventy. Although this age limit has been mentioned over the years, more recent reports do not indicate any increased risk for elderly patients selected for pancreatic surgery[5,11,29].

Surgical techniques of pancreato-duodenectomy for periampullary cancer have changed and unlike Andersen *et al.*, most authors favour the pylorus preserving modification with equal results for survival and possible benefits for weight gain and long term gastrointestinal function[30]. Delayed gastric emptying that has been reported after pylorus preserving pancreatoduodenectomy is only a transient phenomenon and is probably more dependent on the incidence of postoperative complications than on the preservation of the pylorus per se[31].

Andersen *et al.* perform what they called 'extensive lymph node dissections' during PD in this series[1]. According to one Japanese study extended lymph node dissection could result in an improved survival in patients with early stage tumours[32], but prospective studies of the effect of lymph node dissection have not been performed. Extensive dissection around the coeliac axis may cause severe postoperative diarrhoea and, according to our own experience, postoperative septic bleeding probably occurs more frequently after extended lymph node dissection.

Andersen *et al.* show that long term survival was absent in patients with positive para-aortic lymph nodes and that pancreatoduodenectomy was palliative in these patients[1]. Therefore biopsy of pre-aortic lymph nodes with frozen sections could have been helpful in the therapeutic approach during exploratory laparotomy, unless pancreato-duodenectomy is chosen as a palliative procedure. Although diagnostic laparoscopy has proven to be helpful in preoperative staging of pancreatic cancer[33] visualization and biopsy of retropancreatic preaortic region are impossible through the laparoscope.

Survival after resection for periampullary adenocarcinoma in the paper by Andersen *et al.*[1] is in accordance with many other studies and our own experience[34]. Although a five-year survival as high as 50% can be reached after resection for carcinoma of the ampulla, the prognosis for other periampullary tumours is poor and no improvement by the extended lymph node dissection has been achieved in the paper by Andersen *et al.*[1] Adjuvant chemoradiotherapy can be a potential benefit. 5FU with radiotherapy has been shown to be beneficial in one prospective randomized study[35]. The results of two recent prospective trials are awaited.

To conclude: early diagnosis, adequate preoperative conditioning, good preoperative staging including diagnostic laparoscopy, and optimal surgical techniques by an experienced surgeon in a high volume hospital, are all we can offer to these patients to improve short term survival and to lower postoperative morbidity. Whether extended lymph node dissection and perioperative chemotherapy and radiotherapy are beneficial for long term survival should be the subject of further clinical studies.

### REFERENCES

1. Andersen, H.B., Baden, H., Brahe, N.E.B., Burcharth, F. (1994) Pancreaticoduodenectomy for periampullary adenocarcinoma. *J. Am. Coll. Surg.*, **179**, 545–52.

2. Matsumoto, Y., Jujii, H., Miura, K., *et al.* (1992) Sucessful pancreaticojejunal anastomosis for pancreaticoduodenectomy. *Surg. Gynecol. Obstet,* **175**, 555–62.
3. Roder, J.D., Stein, H.J., Huttl, W., *et al.* (1992) Pylorus-preserving versus standard pancreaticoduodenectomy; an analysis of 110 pancreatic and periampullary carcinomas. *Brit. J. Surg.*, **769**, 152–55.
4. Cameron, J.L., Pitt, H.A., Yeo, C.J., *et al.* (1993) One hundred and forty-five consecutive pancreaticoduodenectomies without mortality. *Ann. Surg.*, **217**, 430–38.
5. Hannoun, L., Christophe, M., Ribeiro, J., *et al.* (1993) A report of forty-four instances of pancreaticoduodenal resection in patients more than seventy years of age. *Surg. Gynecol. Obstet.*, **177**, 556–60.
6. Baumel, H., Hugueir, M., Manderscheid, J.C. *et al.* (1994) Results of resection for cancer of the exocrine pancreas; a study from the French Association of Surgery. *Brit. J. Surg.*, **81**, 102–107.
7. Cullen, J.J., Sarr, M.J., Ilstrum, D.M. (1994) Pancreatic anastomotic leak after pancreaticoduodenectomy: incidence, significance, and management. *Am. J. Surg.*, **168**, 295–98.
8. Tsao, J.I., Rossi, R.L., Lowell, J.A. (1994) Pylorus-preserving pancreaticoduodenectomy: is it an adequate cancer operation? *Arch Surg.*, **129**, 405–12.
9. Wade, T.P., Radford, D.M., Virgo, K.S, *et al.* (1994) Complications and outcomes in the treatment of pancreatic adenocarcinoma in the United States veteran. *J. Am. Coll. Surg.*, **1769**, 38–48.
10. Bonenkamp, J.J., Songun, I., Hermans, J., Sasako, M., Welvaart, K., Plukker, J.T.M., Elk, P. van, Obertop, H., Gouma, D.J., Taat, C.W., Lanschot, J. van, Meyer, S., Graaf, P.W. de., Meyenfeldt, M.F. von., Tilanus, H., Velde, C.J.H. van de. (1995) Randomised comparison of morbidity after D1 and D2 dissection for gastric cancer in 996 Dutch patients. *Lancet.*, **345**, 745–48.
11. Lillemoe, K.D. (1995) Current management of pancreatic carcinoma. *Ann. Surg.*, **221**, 133–48.
12. Crile, G. Jr. (1970) The advantages of bypass operations over radical pancreaticoduodenectomy in the treatment of pancreatic cancer. *Surg. Gyn. Obstet.*, **130**, 1049–53.
13. Gordon, T.A., Burleyson, G.P., Tielsch, J.M., Cameron, J.L. (1995) The effects of regionalization on cost and outcome for one general high-risk surgical procedure. *Ann. Surg.*, **221**, 43–50.
14. Lieberman, M.D., Kilburn, H., Lindsey, M., Brennan, M.F. (1995) Relation of perioperative deaths to hospital volume among patients undergoing pancreatic resection for malignancy. *Ann Surg.*, **222**, 638–45.
15. Allema, J.H., Reinders, M.E., Gulik, T.M. van., Leeuwan, D.J. van., Wit, L., Th, de., Verbeek, P.C.M., Gouma, D.J. (1994) Portal vein resection in patients undergoing pancreaticoduodenectomy for carcinoma of the pancreatic head region. *Brit. J. Surg.*, **81**, 1642–46.
16. Reinders, M.E., Allema, J.H., Gulik, T.M. van., Karsten, T.M., Wit, L., Th, de., Verbeek, P.C.M., Rauws, E.J., Gouma, D.J. (1995) Outcome of microscopically non-radical subtotal pancreaticoduodenectomy (Whipple's resection) in the treatment of pancreatic head tumors. *World J Surg.*, **19**, 410–14.
17. Cameron, J.L., Crist, D.W., Sitzman, J.V. *et al.* (1991) Factors influencing survival following pancreaticoduodenectomy for pancreatic cancer. *Am J Surg.*, **161**, 120–25.
18. Marcus, S.G., Cohen, H., Ranson, J.H.C. (1995) Optimal management of the pancreatic remnant after pancreaticoduodenectomy. *Ann Surg.*, **221**, 635–48.
19. Yeo, C.J., Cameron, J.L., Maher, M.M., Sauter, P.K., Zahurak, M.L., Talamini, M.A., Lillemoe, K.D., Pitt, H.A. (1995) A prospective randomized trial of pancreaticogastrostomy versus pancreaticojejunostomy after pancreaticoduodenectomy. *Ann Surg.*, **222**, 580–92.
20. Büchler, M., Friess, H., Klempa, I, *et al.* (1992) Role of octreotide in the prevention of postoperative complications following pancreatic resection. *Am J Surg* **163**, 125–31.
21. Trede, M., Schwall, G. (1988) The complications of pancreatectomy. *Ann Surg.*, **207**, 39–47.
22. Pitt, H.A., Cameron, J.L., Postier, R.G., Gadacz, T.R. (1981) Factors affecting mortality in biliary tract surgery. *Am J Surg.*, **141**, 66–71.
23. Kimmings, A.N., Deventer, S.J.H. van, Obertop, H., Rauws, E.A.J., Gouma, D.J. (1995) Inflammatory and immunologic effects of obstructive jaundice: pathogenesis and treatment. *J Am Coll Surg* **181**, 567–82.
24. Clements WDB, Diamond T, McCoyry, Rowlands BJ. (1993) Biliary drainage in obstructive jaudice: experimental and clinical aspects. *Brit J Surg.*, **80**, 834–42.
25. Gouma, D.J., Coelho, J.C.U., fischer, J.D., Schlegel, J.F., Li, Y.F., Moody, F.G. (1986) Endotoxemia after reilief of biliary obstruction by internal and external drainage in rats. *Am J Surg.*, **151**, 476–79.
26. Lai, E.C.S., Mok, F.P .T., Fan, S.T., Lo, C.M., Chu, K.M., Liu, C.L., Wong, J. (1994) Preoperative endoscopic drainage for malignant obstructive jaundice. *Brit J Surg.*, **81**, 1195–98.
27. Pitt, H.A., Gomes, A.S., Lois, J.F., Mann, L.L., Deutsch, L.S, Longmire, W.P. Jr. (1985) Does preoperative percutaneous biliary drainage reduce operative risk or increase hospital cost? *Ann Surg.*, **201**, 545–53.
28. Berge Henegouwen, M.I., van, Allema, J.H., Gulik, Th.M., Verbeek, P.C.M., Obertop, H., Gouma, D.J. (1995) Delayed massive haemorrhage after pancreatic and biliary surgery. *Brit J Surg.*, **82**, 1527–32.
29. Fong, Y., Blumgart, L.H., Fortner, J.G., Brennan, M.F. (1995) Pancreatic or liver resection for malignancy is safe and effective for the elderly. *Ann Surg.*, **222**, 426–37.
30. Klinkenbijl, J.H.G., Schelling, G.P. vander, Hop, W.C.J., Pel, R. van, Bruining, H.A., Jeekel, J. (1992) The advantages of pylorus-preserving pancratoduodenectomy in malignant disease of the pancreas and periampullary region. *Ann. Surg.*, **216**, 242–45.
31. Yeo, C.J., Barry, M.K., Sauter, P.K., *et al.* (1993) Erythromycin accelrates gastric emptying after pancreaticoduodenectomy: a prospective randomized placebo-controlled trial. *Ann. Surg* **218**, 229–38.
32. Satake, K., Nishiwaki, H., Yokomatsu, H., Kawazoe, Y., Kim, K.A, *et al.* (1992) Surgical curability and prognosis for standard versus extended resection for T1 carcinoma of the pancreas. *Surgery.*, **175**, 259–65.
33. Bemelman, W.A., Wit, L. Th. de, Delden, O.M., van, Smits, N.J., Obertop, J., Rauws, E.J.A., Gouma, D.J. (1995) Diagnostic laparoscopy combined with laparoscopic ultrasonography in staging of cancer of the pancreatic head region. *Brit J Surg.*, **82**, 820–24.
34. Allema, J.H., Reinders, M.E., Gulik, Th. M., Koelemay, M.J., Leeuwen, D.J., van, Wit, L. Th., de, Gouma, D.J, Obertop, H. (1995) Prognostic factors for survival after pancreatoduodenectomy for patients with carcinoma of the pancreatic head region. *Cancer.*, **75**, 2069–76.
35. Moertel, C.G., Frytak, S., Hahn, R.G., *et al.*, (1981) Therapy of locally unresectable pancreatic carcinoma: a randomized comparison of high dose (6000 rads) radiation alone, moderate dose radiation (4000 rads + 5 fluoracil), and high dose radiation + 5 fluoracil) The gastroinstestinal Tumor Study group, *Cancer.*, **48**, 1705–1710.

Amsterdam, 17 January, 1996

H. Obertop, M.D.
D.J. Gouma, M.D.
Department of Surgery
Academic Medical Centre
Meibergdreef 9
1105 AZ Amsterdam
The Netherlands

